# Comparative Genomics Reveals Diversification and Chromosomal Organization of Putative Antimicrobial Peptide-Derived Sequences in Amphibious Mudskippers

**DOI:** 10.3390/biology15161392

**Published:** 2026-08-14

**Authors:** Zhe He, Hanying Wei, Li Deng, Qiong Shi, Chao Bian

**Affiliations:** 1 Laboratory of Aquatic Genomics, College of Life Sciences and Oceanography, Shenzhen University, Shenzhen 518057, China; hezhe011006@gmail.com (Z.H.); 2400171034@mails.szu.edu.cn (H.W.); lideng03@szu.edu.cn (L.D.); 2 Shenzhen Key Lab of Marine Genomics, BGI Academy of Marine Sciences, BGI Marine, Shenzhen 518081, China

**Keywords:** putative AMP-derived sequence, mudskipper, amphibious habitat, comparative genomics, misgurin-like fragment

## Abstract

Antimicrobial peptides (AMPs) and AMP-like fragments are important components of vertebrate innate immunity, but their genome-wide diversification in amphibious fishes remains unclear. Here, we performed integrated bioinformatics and comparative genomic analyses of three representative mudskippers, *Boleophthalmus pectinirostris* (Bp), *Periophthalmus magnuspinnatus* (Pma), and *Periophthalmus modestus* (Pmo), together with zebrafish and humans as the reference vertebrates. Through genomics comparisons, we identified 708 putative AMP-derived genes in the three mudskipper genomes. Compared with zebrafish and humans, mudskippers contained fewer numbers of AMP-derived genes, indicating lineage-associated differences in repertoire size although their evolutionary basis remains unresolved. Several AMP-derived genes showed a pattern of clustered chromosomal distribution, such as histone-associated clusters on the Chr12 and Chr14 of the Bp genome. Histone H2B-derived sequences were highly conserved among various vertebrates, but mudskippers showed a distinct alanine-to-glycine substitution at position 66. In addition, a Misgurin-like fragment was located within *TNNT3a* rather than as an independent AMP gene. Compared with pond loach Misgurin, the synthetic Bp Misgurin-like fragment showed no detectable antibacterial activity against six tested bacterial strains under the tested assay conditions and concentration range, while this fragment was absent from the human *TNNT3* gene. These findings provide new insights into AMP-derived sequence diversity, chromosomal organization, and potential immune adaptation in amphibious mudskippers, and offer candidate resources for future functional validation in medical and aquaculture applications.

## Simple Summary

Mudskippers are unusual fishes that can live both in water and on land, making them useful animals for studying how fishes adapt to challenging environments. In this study, we examined three representative mudskipper species to understand how their natural defense molecules against microbes have changed during evolution. By comparing their genetic information with that of zebrafish and humans, we found many gene regions related to these defense molecules, but mudskippers had fewer such regions. Meanwhile, some of these regions were clustered on specific chromosomes, indicating distinct patterns of genomic organization. We also observed sequence variation in one small defense-related candidate in mudskippers, while the synthesized Bp peptide did not show detectable antibacterial activity under the tested conditions. These results help explain how mudskippers may have adjusted their immune defense while adapting to a transitional life between water and land. Our findings may also provide useful candidate molecules for future studies on development of antibiotic alternatives for aquaculture and clinical practices.

## 1. Introduction

Antimicrobial peptides (AMPs) are short, often cationic and amphipathic peptides that serve as important “offense and defense” molecules in innate immunity [1]. Owing to their broad antimicrobial potential and structural diversity, AMPs have become attractive targets for peptide design, optimization, and nanotechnology-assisted therapeutic development [2]. Among vertebrate AMP-related molecules, histone-derived peptides are particularly noteworthy because histones can function beyond chromatin organization and participate in host defense as antimicrobial agents [3]. The functional characterization of Hipposin, a histone H2A-derived antimicrobial peptide from the mangrove whip ray *Himantura walga*, further shows that conserved intracellular proteins may give rise to AMP-like defense molecules [4]. In fishes, many AMPs have received increasing attention because of their roles in host defense and their potential value as antimicrobial resources for aquaculture and biomedical applications [5]. The recent identification of Bolespleenin334–347, a short cationic peptide from the blue-spotted mudskipper *Boleophthalmus pectinirostris*, further suggests that amphibious fishes may represent underexplored sources of AMPs [6]. Meanwhile, genome-wide identification in catfish and multi-omics screening in the lined seahorse have demonstrated that large-scale omics datasets can expand the discovery of putative AMP-related sequences in aquatic animals [7,8].

Fish live in microbe-rich aquatic environments and rely strongly on innate immune defense, making AMPs important molecular candidates for both immunity research and disease-control applications [9]. With the rapid accumulation of genomic and transcriptomic resources, computational prediction has become an important strategy for identification of AMP candidates from large datasets [10]. The biological relevance of such candidates is supported by functional studies of fish AMPs, as shown by hepcidin-related peptides in olive flounder that exhibit infection-responsive expression, antimicrobial activity, and protective effects against bacterial and viral challenges [11]. More broadly, teleost AMPs are recognized as important mediators of innate immunity and mucosal defense [12]. Classical fish AMPs such as pleurocidin, originally isolated from the skin secretions of winter flounder, highlight epithelial surfaces as important sources of antimicrobial molecules [13]. Building on these biological and computational foundations, high-throughput screening of the Fish-T1K database has accelerated the identification of small immune peptides and AMP candidates across diverse fish lineages [14]. However, genome-scale AMP screening also creates a conceptual challenge: not every sequence homologous to a known AMP is necessarily processed into a mature AMP. Therefore, in this study, “putative AMP-derived sequence” refers to a peptide region homologous to known AMPs or predicted to have AMP-like properties, while “AMP-derived gene” refers to the protein-coding gene containing such an AMP region. Because many predicted regions are embedded within larger proteins, they should be considered as candidate AMP-like fragments while requiring future functional validation rather than confirmed mature AMPs. Therefore, some putative AMP-derived sequences may represent cryptic antimicrobial peptides embedded within larger proteins before being released through proteolytic cleavage.

Mudskippers are amphibious teleost that inhabit intertidal environments and represent valuable models for studying adaptation to a transitional life between water and land. Previous studies in *Boleophthalmus pectinirostris* have identified immune-related genes such as hepcidins, indicating that this mudskipper species possesses molecular components involved in host defense [15]. At the genome level, mudskipper genomes have provided important clues to the genetic basis of terrestrial adaptation in amphibious fishes [16]. Their water-to-land lifestyle is also supported by specialized morphological traits, including respiratory vasculature adapted to variable aquatic and terrestrial environments [17]. Functional studies of *B. pectinirostris* hepcidins and NK-lysin further suggest that peptide-mediated immune defense may be important for mudskippers living in microbially complex intertidal habitats [18,19]. Taxonomically, mudskippers belong to oxudercine gobies, a diverse group that includes ecologically differentiated lineages such as *Boleophthalmus*, *Periophthalmus*, and *Periophthalmodon* [20]. In this study, we selected three representative mudskippers, *B. pectinirostris*, *Periophthalmus magnuspinnatus*, and *Periophthalmus modestus*. These species differ in habitat preference, with aquatic dependence generally following Bp > Pmo > Pma. In addition, chromosome-level genome assemblies are available for them, providing a consistent genomic framework for comparing the diversity and chromosomal organization of predicted AMP-derived sequences.

Despite these advances, the genome-wide organization and evolutionary diversification of mudskipper AMP-derived sequences remain insufficiently resolved. Previous high-throughput screening identified AMP candidates from amphibious mudskippers, providing an important starting point for AMP discovery in this group [21]. Broader comparative genomics studies of ray-finned fishes have constructed a framework for interpreting fish genome divergence, evolution, and adaptation [22], while proteomic analysis of Bp skin mucus suggested that epidermal secretions may contribute to host defense in intertidal environments [23]. More recently, chromosome-level genome comparisons of three mudskipper species revealed molecular clues related to water-to-land evolution and adaptation, supplying a genomic basis for chromosome-scale analyses of AMP-derived sequences [24]. Additional studies on mudskipper genetic adaptation, fish gene-family evolution, and convergent water-to-land transition further support mudskippers as useful models for studying environmental adaptation [25,26,27]. In parallel, AMP-related genomic and functional studies in other teleost, including giant grouper (*Epinephelus lanceolatus*), redbanded seabream (*Pagrus auriga*), and Caspian trout (*Salmo trutta caspius*), have demonstrated the feasibility of identifying and interpreting AMP-related genes through comparative and functional approaches [28,29,30]. Genome-wide chromosome-level mapping allows AMP-derived genes to be examined in their genomic context, including whether they are dispersed or clustered across diverse chromosomes. This allows for direct comparison of whether AMP-derived genes show similar or distinct chromosomal distribution patterns among the three mudskipper species. However, a chromosome-scale comparative analysis focused on AMP-derived sequences in amphibious mudskippers remains lacking.

Here, we employed chromosome-level genome assemblies of three representative mudskippers, together with zebrafish and human reference genomes, to screen putative AMP-derived sequences and map their corresponding source genes. We compared category composition, copy number patterns, genome-wide distribution, Misgurin-like sequence variation, and histone-derived AMP-like fragments across these examined vertebrates, with particular attention to sequence variation at residue 66 of histone H2B. We also synthesized the Bp Misgurin-like fragment and tested its antibacterial activity against six popular bacterial strains. This study provides candidate resources and a comparative evolutionary framework for future functional validation, rather than direct evidence that all predicted fragments act as mature AMPs.

## 2. Materials and Methods

### 2.1. Data Collection

A total of 3940 antimicrobial peptide sequences were collected from the Antimicrobial Peptide Database (APD3, https://aps.unmc.edu/, accessed on 20 May 2025) [31] to construct a reference database for screening putative AMP-derived sequences in three mudskipper species, zebrafish, and humans. Genome data of the three representative mudskippers were obtained from our previous report [24] and the National Center for Biotechnology Information (NCBI, https://www.ncbi.nlm.nih.gov/, accessed on 1 August 2025) database: *Boleophthalmus pectinirostris* genome (Bp, ASM2622593v1, GCF_026225935.1), *Periophthalmus magnuspinnatus* genome (Pma, ASM2622583v1, GCF_009829125.3), and *Periophthalmus modestus* genome (Pmo, ASM1959493v1, GCA_019594935.1). The genomes of zebrafish and humans were also downloaded from the NCBI database with accession numbers GCF_000002035.6 (zebrafish *Danio rerio*, GRCz11) and GCF_000001405.40 (human *Homo sapiens*, GRCh38.p14).

Amino acid sequences of histone H2B, a representative source protein frequently associated with histone-derived AMP-like fragments, were downloaded from NCBI for multiple sequence alignment and phylogenetic analysis. Seven representative vertebrate species, including striped bass (*Morone saxatilis*, XP_035526269.1), southern bluefin tuna (*Thunnus maccoyii*, XP_042285779.1), African clawed frog (*Xenopus laevis*, XP_018120357.1), plains spadefoot toad (*Spea bombifrons*, XP_053323647.1), Asiatic toad (*Bufo gargarizans*, XP_044129366.1), mouse (*Mus musculus*, NP_001300807.1), and chimpanzee (*Pan troglodytes*, XP_054511317.2) were examined. Other relevant sequences were obtained by processing these genomes with custom Perl scripts. In addition, the human TNNT3 (troponin T type 3, fast skeletal) protein (XP_016873696.1) and the pond loach (*Misgurnus anguillicaudatus*; abbreviated as Ma) *TNNT3a* transcript (XM_055193150.1) were downloaded from NCBI, and Misgurin-like target regions were compared with the Bp TNNT3a sequence to identify sequence differences among the examined taxa.

### 2.2. Prediction and Identification of Putative AMP-Derived Sequences

The screening workflow was adapted from previously reported high-throughput AMP identification strategies with minor modifications [21,32,33]. In brief, APD3-derived AMP sequences [31] were used as a query library, and local genome, gene set, and transcriptome databases of the five examined vertebrates (i.e., Bp, Pma, Pmo, zebrafish, and human) were constructed using the makeblastdb command in BLAST v2.6.0 [34]. TBLASTN searches were then performed against these local databases with an E-value threshold of 1 × 10^−5^. Hits were retained when their aligned region covered at least 50% of the corresponding AMP query sequence. For multiple matches to the same subject sequence, alignments were ranked by sequence identity and only the best-matching record was retained. Based on the locus information in the alignment results, corresponding coding sequences (CDSs) were extracted and deduced into amino acid sequences with custom Perl scripts, yielding candidate AMP-derived peptide regions.

AMPml v1.2.7 (https://github.com/flystar233/AMPml, accessed on 1 May 2025), an AMP prediction tool written in Python v3.14.2 that integrates four machine-learning algorithms and three amino acid feature descriptors, was then applied to further evaluate these candidate sequences. A probability threshold of 0.5 was used for AMP classification, with sequences having an AMP probability ≥ 0.5 retained as putative AMP candidates. These candidates were then linked to their corresponding APD3 query sequences to assign preliminary AMP-related categories. For those candidates with unclear category assignments, BLASTP searches were performed using NCBI BLAST+ v2.17.0 against the NCBI database were performed to identify their source protein categories and improve annotation. After category assignment, the numbers of AMP-derived peptide regions and corresponding source genes were counted for each AMP-related category. For the three mudskipper species, different categories of putative AMP-derived sequences were separately aligned using BioEdit v7.0.9 [35]. Incomplete genes or transcripts were removed, and only reliable candidate sequences were retained to establish a mudskipper putative AMP-derived sequence dataset.

### 2.3. Comparative Genomic Analysis of AMP-Derived Genes

Comparative genomic analyses were performed to examine differences in putative AMP-derived sequences and their corresponding source genes among the five examined vertebrates. The analyzed features included AMP-related category composition, total numbers of candidate peptide regions, source gene numbers, species-shared and species-specific categories, and copy number variation of representative AMP-derived genes [21,28,36]. Candidate categories or source genes showing lineage-specific patterns were further examined to assess their possible association with amphibious adaptation. Because most sequences were predicted from genomic data, functional interpretations were treated as hypotheses requiring experimental validation rather than direct evidence of mature AMP.

### 2.4. Genome-Wide Distribution of AMP-Derived Genes

Based on the genomic coordinates of AMP-derived source genes, TBtools-II v2.303 (https://github.com/CJ-Chen/TBtools, accessed on 1 May 2025) was employed to map their positions onto the corresponding chromosomes of the three mudskipper species [37]. The chromosomal locations of representative AMP-derived genes and gene clusters were marked following genome-wide analyses in fishes, providing a genomic framework for subsequent comparative and functional analyses [38].

### 2.5. Phylogenetic Analysis of Histones and Histone-Derived AMP-like Fragments

Multiple sequence alignments were conducted for putative AMP-derived peptide sequences together with reference AMP sequences from known species using MAFFT v7.037 [39]. The aligned sequences were sorted according to sequence similarity and visualized using TexShade v1.29 to highlight conserved residues at identical positions [40]. Amino acid sequences of histone proteins and histone-derived AMP-like fragments from the three mudskippers were further aligned using MUSCLE v3.8.31 [41]. Phylogenetic trees of mudskipper histones and related histone-derived proteins were constructed using the maximum-likelihood method in MEGA 11 [42].

### 2.6. Physicochemical Characterization and Structural Prediction of Putative AMP-Derived Peptides

To analyze the physicochemical properties of putative AMP-derived peptides, amino acid composition, net charge at physiological pH, and isoelectric point were calculated using the pepstats tool within the EMBOSS-6.6.0 suite [43]. Schiffer–Edmundson helical wheel projections, hydrophobicity profiles, and hydrophobic moment values were generated using the HeliQuest web server (https://heliquest.ipmc.cnrs.fr/cgi-bin/ComputParamsV2.py, accessed on 20 May 2025) to evaluate potential membrane-interaction properties [44]. Tertiary structure predictions were performed using AlphaFold3 with default parameters [45]. Secondary structure elements were annotated using the PredictProtein platform (https://www.predictprotein.org/, accessed on 1 May 2025), an online resource for protein structural and functional feature prediction [46]. Structural alignments and molecular visualizations were subsequently conducted in PyMOL v3.1.0 to compare conformational features [47].

### 2.7. Synthesis and Antimicrobial Activity Assessment of the Bp Misgurin-like Peptide

The predicted Bp Misgurin-like peptide fragment was synthesized using Fmoc solid-phase peptide synthesis followed by resin cleavage [48]. Peptides were purified using an analytical HPLC system (Kromasil 100-5-C18 column, 4.6 × 250 mm; AkzoNobel, Bohus, Sweden) under a 1 mL·min^−1^ acetonitrile gradient with Buffer A consisting of 0.1% trifluoroacetic acid (TFA) in acetonitrile and Buffer B consisting of 0.1% TFA in H_2_O. Peptides with >95% purity, confirmed by HPLC-MS/MS, were commercially synthesized by GL Biochem Ltd. (Shanghai, China). The molecular weight of the synthesized Bp Misgurin-like peptides was 2501.97 Da. They were reconstituted in sterile deionized water, and then stored at −80 °C before use.

Antibacterial activity of the synthesized Bp Misgurin-like peptide was evaluated against six bacterial strains, including Streptococcus agalactiae (GDMCC 1.408), Salmonella enteritidis (GDMCC 1.163), Staphylococcus aureus (GDMCC 1.1220), Escherichia coli (GDMCC 1.137), Aeromonas hydrophila (GDMCC 1.2306), and Vibrio anguillarum (GDMCC 1.2067), following a previously described AMP activity assay with modifications [49]. Logarithmic-phase bacterial cultures were washed three times with phosphate-buffered saline (PBS), resuspended to 10^4^ CFU·mL^−1^, and incubated with peptide solutions for 1 h at room temperature. Bacterial cultures without peptide treatment were used as growth controls, and PBS or medium without bacteria was used as blank controls. The mixtures were then transferred to 96-well plates containing 200 μL of LB medium. Initial screening was performed at 39.97 μmol·L^−1^ (equivalent to 100 μg·mL^−1^), and minimum inhibitory concentration (MIC) values were determined using serial peptide concentrations of 2.5, 5, 10, 20, and 40 μmol·L^−1^. Plates were incubated in a microplate reader (Biotek, Winooski, VT, USA) at 28 °C for Aeromonas hydrophila and Vibrio anguillarum, and at 37 °C for Streptococcus agalactiae, Salmonella enteritidis, Staphylococcus aureus, and Escherichia coli, with 5-s orbital shaking intervals. OD600 values were measured at 2, 4, 8, 12, and 20 h over a 20 h period to generate bacterial growth curves. Experiments were repeated three times for each strain.

## 3. Results

### 3.1. Diversity and Representative Types of AMP-Derived Genes in the Three Mudskippers

**Figure 1:**
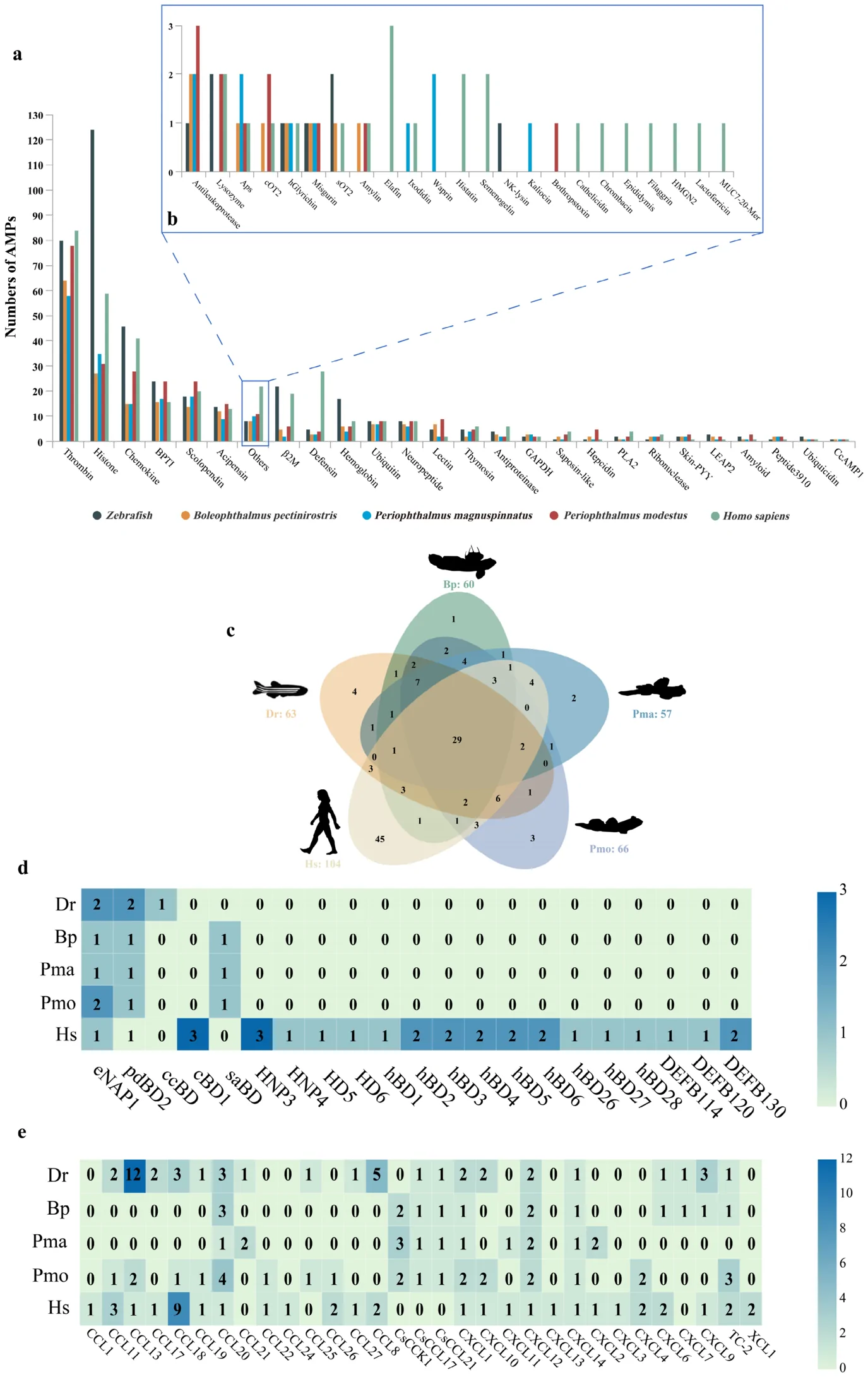
Comparative distribution of AMP-derived genes in the five examined species. (**a**) Summarized numbers of AMP-derived genes in the five vertebrate species. (**b**) Within the category of “others”, at least one of the five species did not have a specific AMP-derived gene. (**c**) A Venn diagram of AMP-derived gene numbers in the five examined species (see more details in Table 1). (**d**) Number of Defensins and its derived peptides in the five examined vertebrates. (**e**) Number of Chemokines and its derived peptides in the five examined vertebrates.

After sequence alignment and manual verification, we identified 406, 215, 208, 285, and 360 putative AMP-derived genes from the genomes of five representative vertebrates, including fully aquatic zebrafish (Dr: *Danio rerio*), three amphibious mudskippers (Bp, Pma, Pmo), and terrestrial humans (Hs: *Homo sapiens*), respectively (see more details in Table 1). The total numbers of AMP-derived genes were broadly similar among the three mudskipper species, with Pmo showing a slightly higher number than Bp and Pma, but lower than zebrafish and humans, with obvious differences of several tens to over one hundred genes (Figure 1 and Table 1).

Thrombin-associated source genes containing C-terminal AMP-like regions were the most numerous, consistent with the antimicrobial roles of thrombin-derived host-defense peptides in treatment of wounds [50]. Striking interspecific divergence emerged among histone-derived categories, which represented the most variable group among the 48 detected AMP-derived categories in this study. Given the reported antimicrobial and immunomodulatory roles of histones and histone-derived peptides [51], we further performed phylogenetic analysis of these histones and their derived fragments in mudskippers as described in Section 3.4. Histone-, chemokine-, and β2-microglobulin (β2M)-derived categories showed comparable counts among mudskippers but were reduced compared with zebrafish and humans (Figure 1a), suggesting lineage-specific differences in AMP-derived genes associated with innate immune defense and mucosal protection in fish [52]. Notably, as an important AMP representative, Misgurin-like fragments were detected in the four examined fish genomes but not in the corresponding human TNNT3 region, suggesting lineage-specific divergence of the TNNT3 C-terminal region (see more details in Section 3.3). In contrast, ten AMP-derived genes appeared exclusively in humans, while no homologous sequences were identified in the examined fish species (Figure 1b).

Unlike thrombin, which is the most numerous among AMP-derived source genes, chemokines represent the most diverse and complex family among all AMP category families, with up to 25 different chemokine-derived peptide sequences in humans (Figure 1e). Chemokines mediate immune-cell migration and localization and play important roles in innate immune responses [53]. Fish defensins are stable antimicrobial peptides with important roles in teleost host defense [54]. Notably, defensin-derived genes were fewer in the four fish species (3~5 genes) than in humans (28 genes) (Figure 1d). As illustrated in Figure 1c, the total AMP-derived categories in zebrafish and the three mudskipper species are generally similar (~60), with only 4, 1, 2, and 3 species-specific AMP-derived categories, respectively. Compared to the 45 species-specific AMP-derived categories in humans, the difference is substantial. These interesting differences suggest lineage-specific diversification of AMP-derived categories and source genes in humans.

### 3.2. Genome-Wide Distribution of AMP-Derived Genes in the Three Mudskipper Species

**Figure 2:**
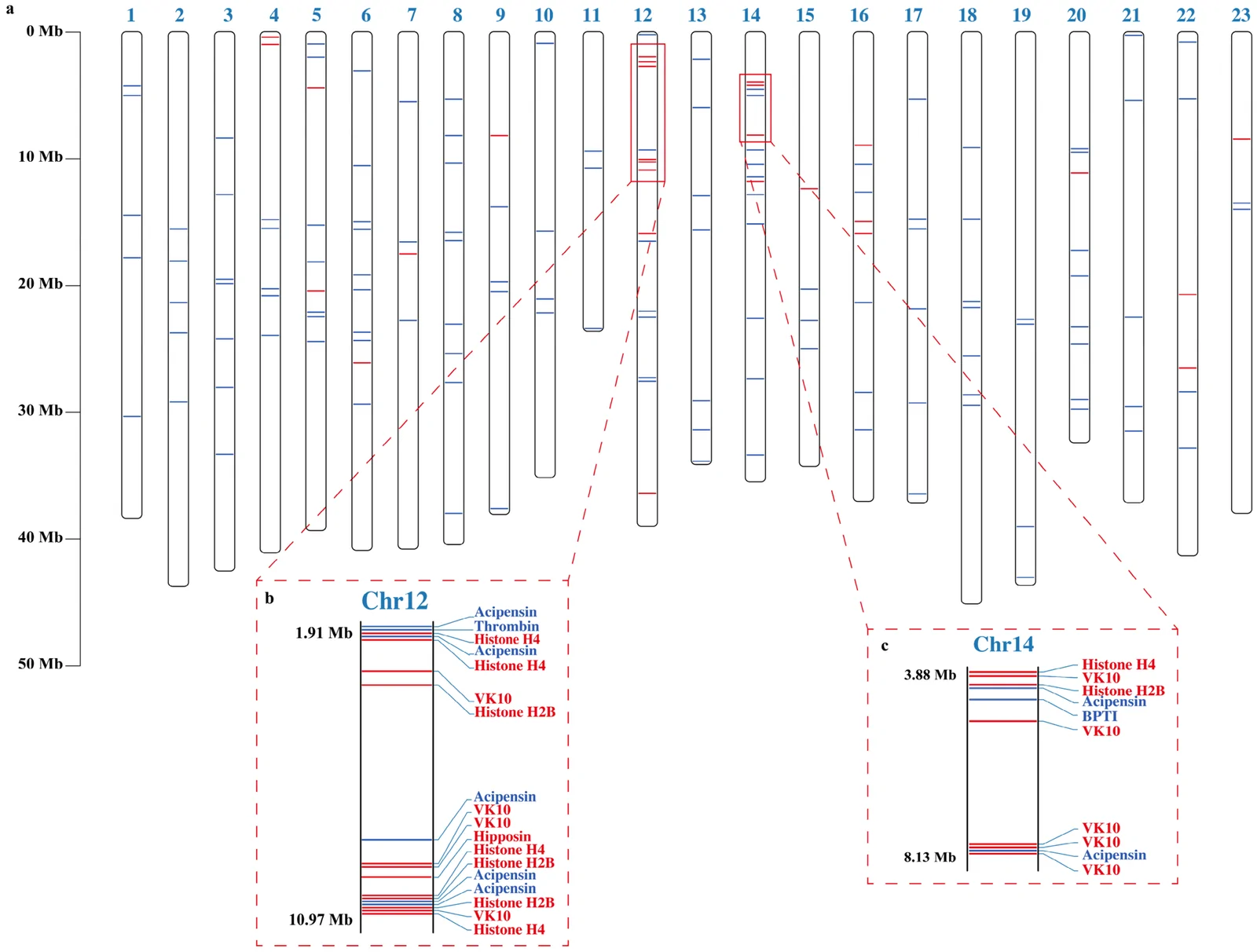
Genome-wide distribution of AMP-derived genes in Bp. (**a**) The serial numbers at the top represent chromosome ID. The haplotypic 23 chromosomes were assembled and annotated. Red bars represent histone-derived AMP-containing genes, while blue bars stand for non-histone-derived AMP-containing genes. (**b**) A total of twelve histone-derived AMP-containing genes are clustered on Chr12. (**c**) Seven histone-derived AMP-containing genes are clustered on Chr14.

Comparative chromosomal mapping of AMP-derived genes across the three mudskipper genomes revealed several gene clusters and conserved lineage-specific genomic clustering patterns. For instance, in Bp, a total of 215 AMP-derived genes were localized across the 23 haploid chromosomes (Chr; Figure 2a), with two prominent histone-associated clusters within Chr12 (Figure 2b) and Chr14 (Figure 2c). These clusters contained twelve and seven histone-derived AMP-containing genes, respectively, predominantly encoding H4, H2B, and VK10 (a histone-derived peptide first identified from *Varanus komodoensis* [55]). Notably, these gene clusters also contained acipensin-like sequences, such as acipensin 6, a histone H2A-derived antimicrobial fragment spanning residues 62–85 that was classified separately from the histone-derived category [56], alongside bovine pancreatic trypsin inhibitor (BPTI) and thrombin homologs.

Pma had 208 AMP-derived genes dispersed across the total of 25 chromosomes (Figure S1a), an expanded karyotype with two more chromosomes than Bp and Pmo via chromosomal fission [24]. Histone-derived AMP-containing genes clustered on Chr14 (eight genes; Figure S1b) and Chr19 (seven genes; Figure S1c), encompassing four types of histones and histone-derived peptides (H4, H3, H2A, and H2B) as well as ubiquitin-derived peptides. These clusters included ubiquitin-derived peptide regions, which have been proven to contribute to antimicrobial activity [57].

Similarly, Pmo harbored 285 AMP-derived genes across 23 chromosomes (Figure S2a), featuring clusters on Chr8 (eight genes; Figure S2b) and Chr21 (twelve genes; Figure S2c) that included Buforin I (derived from H2A N-terminal domain), which can further generate Buforin II, a peptide with even higher antimicrobial activity [58]. Histone-derived peptides are important AMP-like molecules involved in innate immunity and bacterial killing mechanisms [59,60]. These intriguing phenomena of gene clustering may represent an evolutionary response of mudskippers to the environmental pressures associated with amphibious habitats (see more discussions in Section 3.4).

### 3.3. Evolutionary and Functional Characterization of Misgurin from Different Species

**Figure 3:**
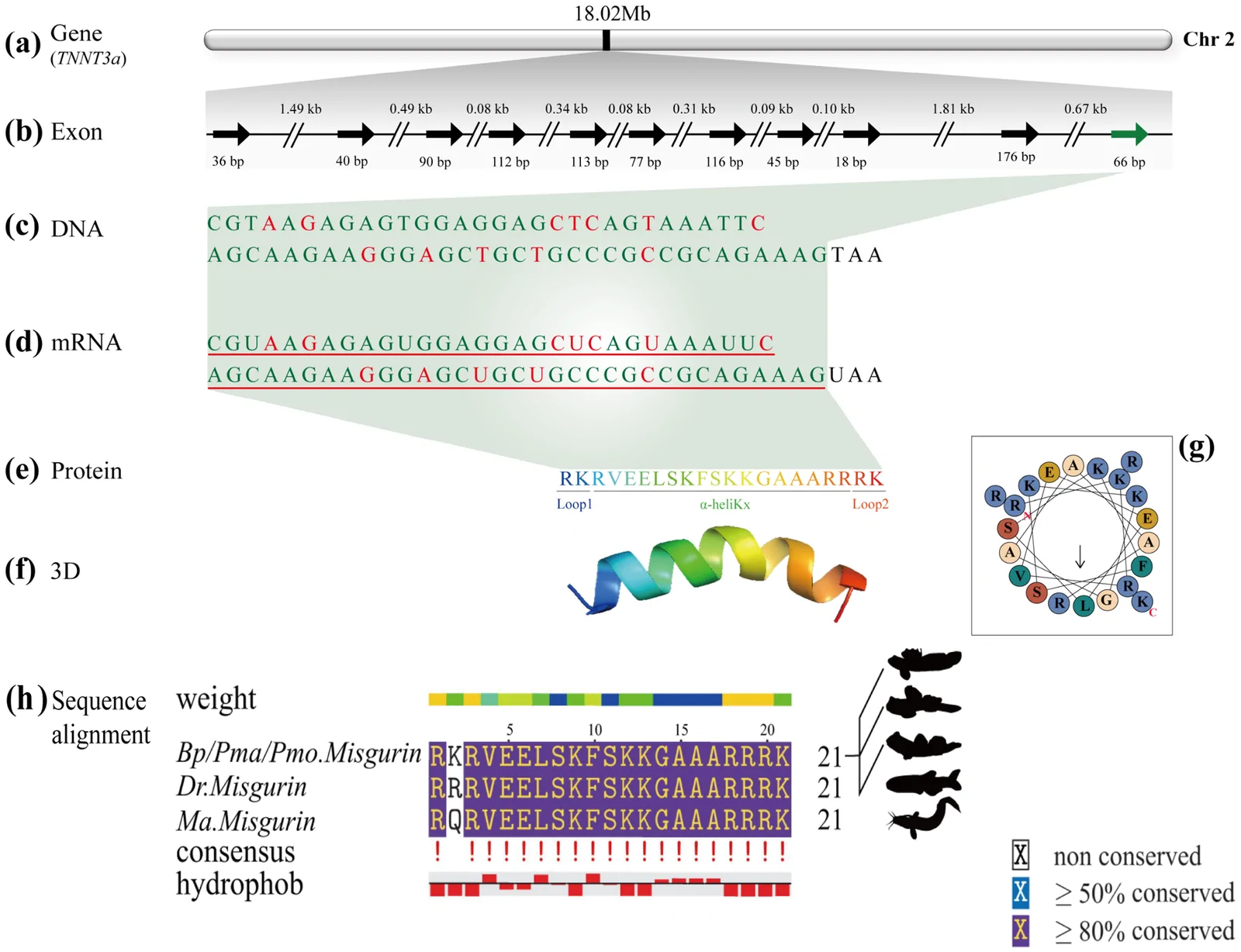
Characterization of the representative Misgurin and its derived gene (*TNNT3a*) in the Bp genome. The Bp Misgurin-like fragment is located in exon 11, the terminal exon of the annotated *TNNT3a* gene (**a**), marked in green. Double slashes represent introns (**b**), the red letters represent those bases that differ (**c**) between the two species (Ma and Bp), and the red line represents the nucleotide sequence encoding Misgurin (**d**). Deduced protein sequence (**e**) and its predicted three-dimensional structure (**f**,**g**) are provided for comparison. Peptide sequences of Misgurin in the representative bony fishes (**h**) are aligned to reveal conservation and variance.

**Figure 4:**
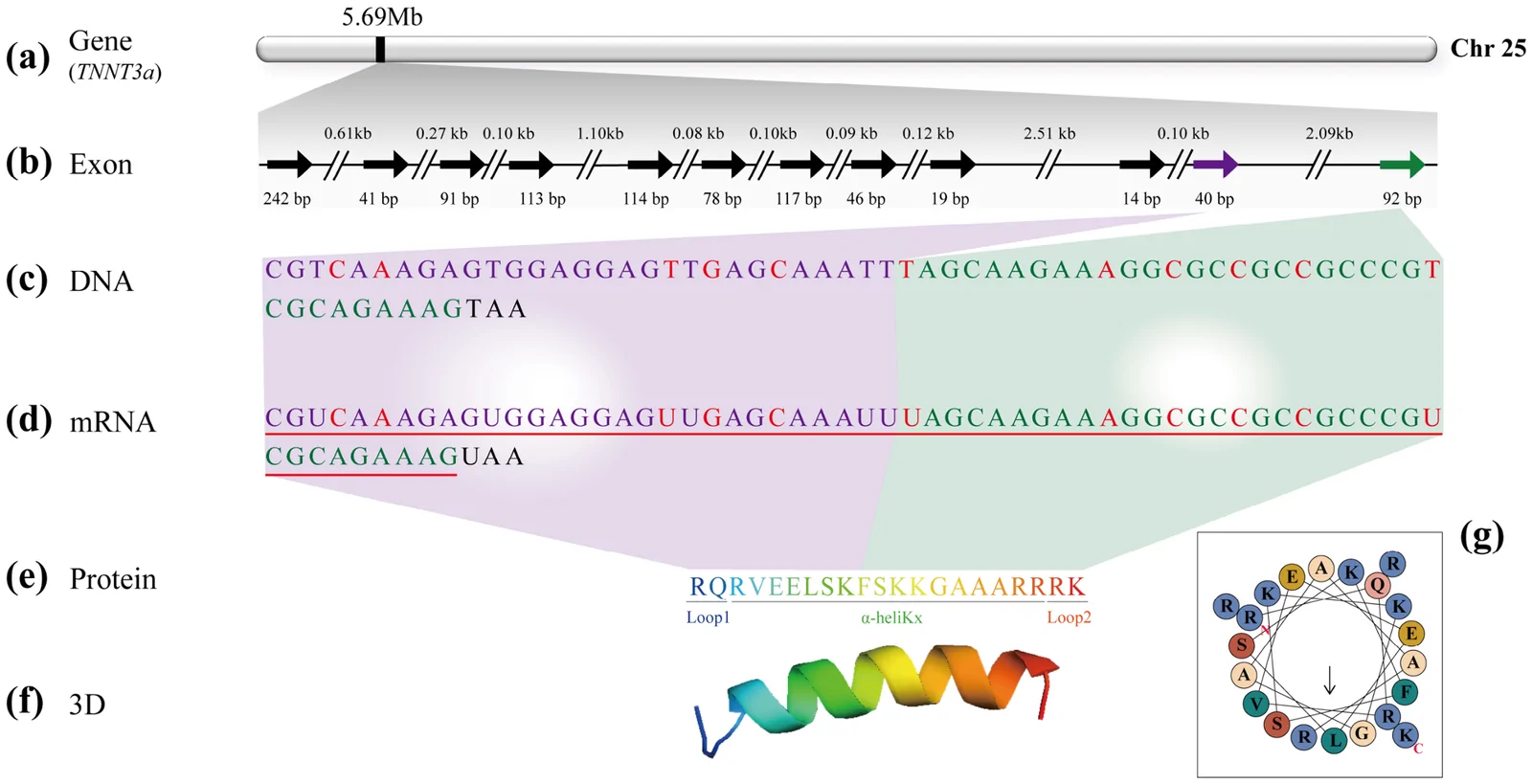
Characterization of the representative Misgurin and its derived gene (*TNNT3a*) in the Ma (*Misgurnus anguillicaudatus*) genome. The Ma Misgurin sequence is located in exons 16 and 17 of the *TNNT3a* gene (**a**), marked in purple and green, respectively. Double slashes represent introns (**b**), the red letters represent those bases that differ (**c**) between the two species (Ma and Bp), and the red line represents the nucleotide sequence encoding Misgurin (**d**). Deduced protein sequence (**e**) and its predicted three-dimensional structure (**f**,**g**) are provided for comparison.

The representative Misgurin-like fragment was located within the *TNNT3a* gene on Bp Chr2 (Figure 3 and Figure 4). *TNNT3a* encodes fast skeletal muscle troponin T, a protein involved in vertebrate striated muscle contraction [61]. Misgurin usually comprises 21 amino acid residues (aa), forming a single cysteine-free α-helix (17 aa).

**Figure 5:**
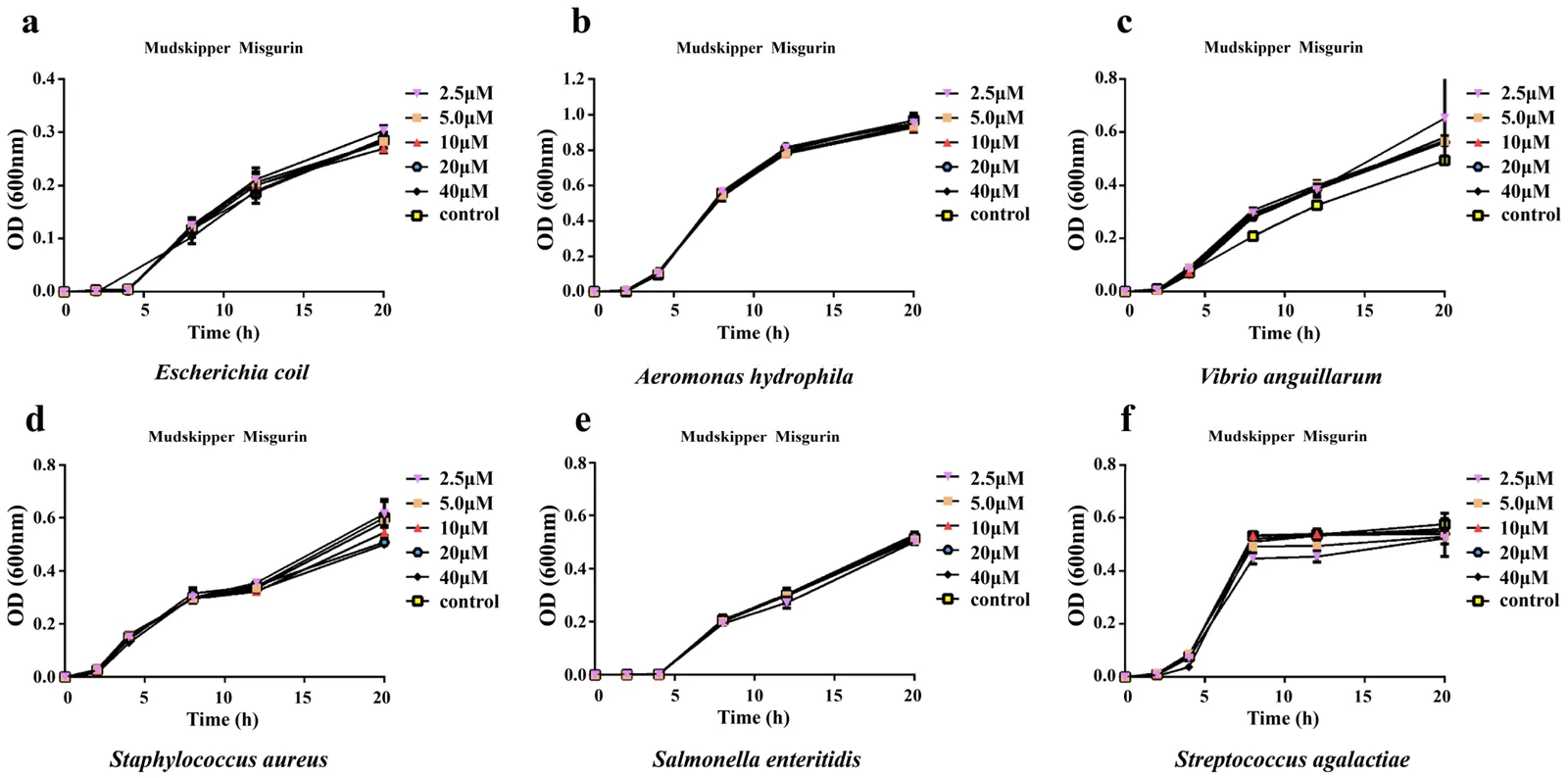
Antibacterial activity assessment of the Bp Misgurin-like peptide against six bacterial strains over 20 h. (**a**) *Escherichia coli*; (**b**) *Aeromonas hydrophila*; (**c**) *Vibrio anguillarum*; (**d**) *Staphylococcus aureus*; (**e**) *Salmonella enteritidis*; (**f**) *Streptococcus agalactiae*.

Misgurin was initially identified in pond loach *Misgurnus anguillicaudatus* and exhibited broad-spectrum antibacterial activity in vitro (Table S1) without hemolysis (Table S2) [62]. Therefore, the Ma *TNNT3a* region containing the native Misgurin was used as a reference for comparison with the Misgurin-like fragment identified in Bp *TNNT3a*. Sequence comparison showed that Bp, Pma, and Pmo contain lysine (K) at residue 2 of the Misgurin-like fragment, whereas Ma Misgurin contains glutamine (Q) at the corresponding position (Figure 3e,h and Figure 4e). The synthetic Bp Misgurin-like peptide showed no detectable antibacterial activity against the six examined bacterial strains under the tested assay conditions and concentration range (Figure 5).

Further structural analysis revealed nearly identical α-helical topology (Figure 3h), tertiary structure (Figure 3f and Figure 4f), and physicochemical properties (Table S3) between the two variants. However, the aforementioned results do not directly refute the rationality of the screening process for putative AMP-derived sequences. It remains possible that this fragment may show activity against untested microorganisms or under different assay conditions. For instance, if the concentration range of the Bp Misgurin-like fragment in antimicrobial assays is further extended, activity at higher concentrations cannot be excluded, but antibacterial effects requiring very high peptide concentrations may have limited practical applicability.

Furthermore, a comparative analysis of the whole-genome data from the five compared species revealed that with the exception of humans (Figure S3), Misgurin-like sequences were detected in the four examined fish genomes (for example, the Bp Misgurin-like fragment is located in exon 11, the terminal exon of the annotated *TNNT3a* gene; see Figure 3b). We thereby hypothesize that during the evolutionary adaptation for aquatic organisms with partial terrestrial life, the AMP sequences within *TNNT3a* genes underwent certain modifications. Consequently, we conducted a sequence comparison between the Bp *TNNT3a* gene (Figure 3) and human *TNNT3* (Figure S3). We compared the corresponding TNNT3 C-terminal regions of the missing Misgurin sequence in the human *TNNT3* gene, and visualized this differential detail in Figure S3. In brief, our findings indicate that the *TNNT3* gene is located on Chr11 of the human genome and comprises 17 exons (Figure S3a,b). The sequence corresponding to Misgurin in the human *TNNT3* gene should be located on exons 16 and 17, encoding 27 amino acids (Figure S3e). However, it has been changed (no longer being Misgurin), with an α-helical structure (formed by eight amino acid residues) and additional residue-formed loops.

### 3.4. Phylogeny of Histones and Their Derived Genes in the Three Mudskippers

**Figure 6:**
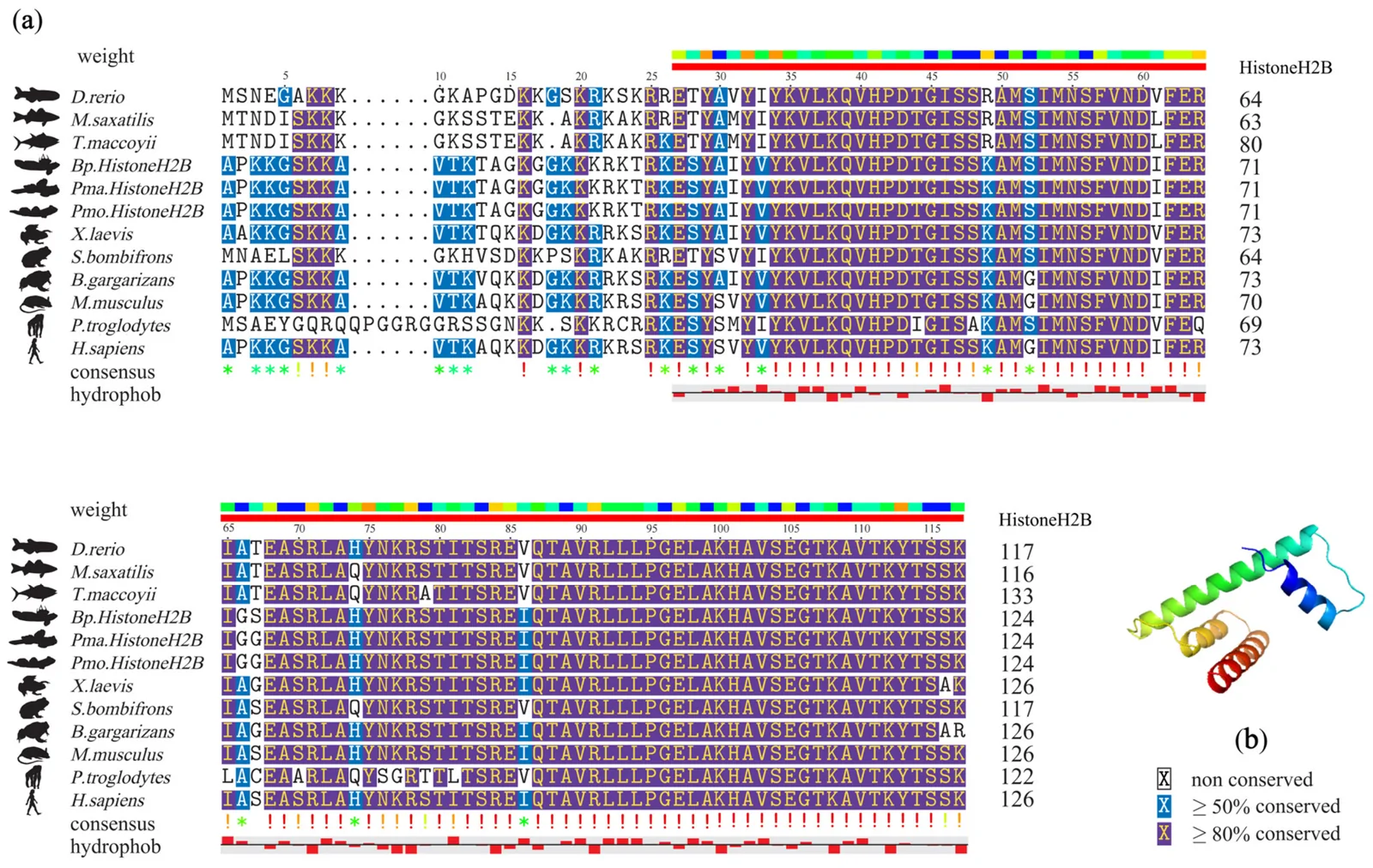
Multiple sequence alignment (**a**) and structural prediction (**b**) of Histone H2B in various vertebrates. (**a**) Red line marks the fragment of Histone H2B within the protein sequences, indicating the high conservation of histone H2B among different vertebrate species. (**b**) Blue region is more than 50% conserved, and purple region is more than 80% conserved. Weight indicates the molecular weight of a protein, and consensus indicates the similarity of protein sequences among different species. Hydrophobe means hydrophobic property: upper red boxes represent hydrophobic residues, while lower red boxes represent hydrophilic residues.

We conducted a multiple sequence alignment of histone H2B proteins across twelve vertebrate species, including three typical aquatic fishes (zebrafish: *D. rerio*, striped bass: *M. saxatilis*, southern bluefin tuna: *T. maccoyii*), three amphibious mudskippers (Bp, Pma, and Pmo), three amphibious toads (African clawed frog: *X. laevis*, plains spadefoot toad: *S. bombifrons*, Asiatic toad: *B. gargarizans*), and three mammals (chimpanzee: *P. troglodytes*, mouse: *M. musculus*, human: *H. sapiens*). These species provide a broad representation of vertebrates with aquatic, amphibious, and terrestrial lifestyles. A phylogenetic analysis revealed strong evolutionary conservation of Histone H2B sequences among all groups (Figure 6a), likely due to the universally important role of histones in the immune defense of various vertebrates. Nevertheless, there are differences in Histone H2B sequences among diverse species to varying degrees. For example, mudskippers showed a glycine residue at position 66, whereas alanine was conserved in the other examined vertebrates. In addition, structural modeling predicted four α-helices in Bp Histone H2B (Figure 6b), which is consistent with the canonical histone architecture.

**Figure 7:**
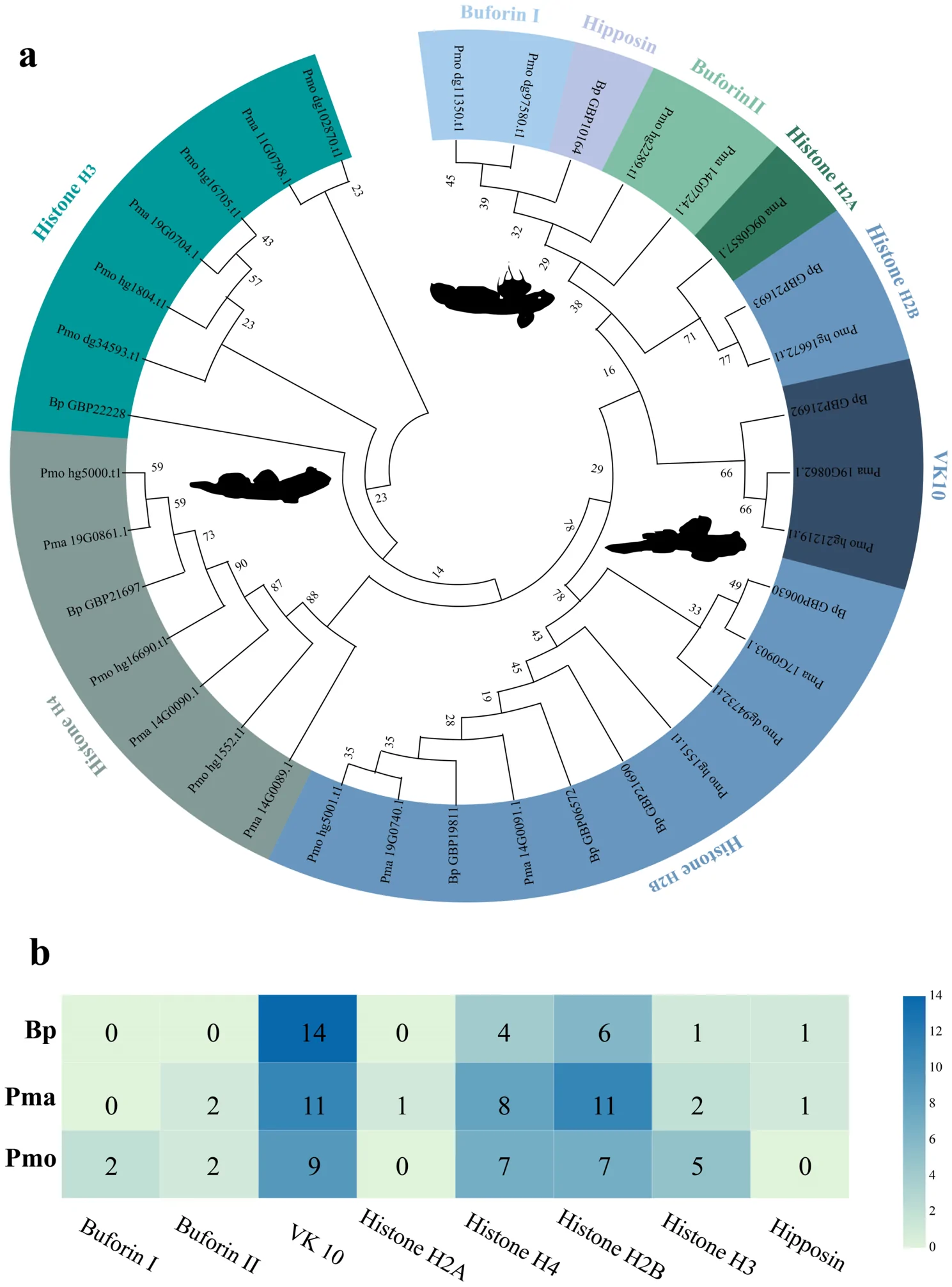
The number and evolutionary relationship of Histones and histone-derived peptides in the three mudskipper species. (**a**) Phylogenetic analysis of 35 non-redundant histone and their derived peptide sequences. (**b**) Number of Histones and histone-derived peptides in the three mudskippers.

Histone-related AMP-derived categories were prominent in the mudskipper genomes. A comprehensive screening of the three mudskipper genomes identified 94 histones and histone-derived peptides (Figure 7), such as Hipposin, Buforin I, and Buforin II (each with two copies), and VK10, the most abundant category, with 34 copies in total across the three mudskippers (14 in Bp, 11 in Pma, and 9 in Pmo; Figure 7b). Among the three mudskipper species, the combined numbers of Histone H2B, H4, H3, and H2A were 24, 19, 8, and 1, respectively (Figure 7b). The low count of Histone H2A may arise from two factors: (1) Buforin I and Hipposin, derived from the N-terminal domain of Histone H2A, are classified as histone-derived peptides [4,58]; (2) Acipensin, an antimicrobial fragment spanning residues 62–85 of histone H2A, was classified separately from histone-derived categories [56].

In the phylogenetic analysis of 35 non-redundant histone and histone-derived peptide sequences from the three mudskipper species (Figure 7a), we found that Hipposin, Buforin I, Buforin II, and Histone H2A, all belonging to the Histone H2A-derived group, were most closely related phylogenetically. Within Histone H2B, several AMP fragments with significant evolutionary divergence were identified. Notably, VK10 is homologous to Histone H3 in the Komodo dragon [55], but in mudskippers it is more closely related to Histone H2B.

## 4. Discussion

Mudskippers, a unique group of amphibious fishes, exhibit distinct AMP-derived sequence diversity compared with those species restricted to either fully aquatic or terrestrial habitats. In this study, we identified 708 putative AMP-derived genes from three representative mudskipper species and compared them with those in zebrafish and humans through comparative genomic analyses. The three mudskipper genomes showed comparable numbers of AMP-derived genes, but contained fewer genes with putative AMP-derived fragments than in zebrafish and humans. This difference may result from lineage-specific gene gain or loss, genetic drift, altered gene duplication rates, or variation in genome assembly and annotation completeness. Previous studies have identified multiple instances of adaptive molecular evolution in amphibious fishes during adaptation to aquatic and terrestrial environments [63]. Thus, the possibility that some AMP-derived regions were lost or remodeled during this water-to-land transition cannot be excluded, although this hypothesis requires further investigation. In humans, the larger number of AMP-derived categories and source genes may reflect lineage-specific diversification of immune-related sequence repertoires. More broadly, positive selection has been reported for vertebrate AMP genes [64], although its relevance to the pattern observed here needs more explorations. To explore these patterns further, in this study we focused on the Misgurin-like fragment, which was absent from the human *TNNT3* region, and on histone-related AMP-derived categories that showed remarkable interspecific variation.

The Misgurin-like fragment identified in mudskippers was located in the carboxyl-terminal region of the *TNNT3a* gene rather than in a standalone AMP gene. Fish skeletal muscle, similar to skin, can function as an immune-active tissue during pathogen infection [65]. In humans, however, the corresponding Misgurin-like fragment is absent from the TNNT3 region, which may reflect lineage-specific sequence remodeling rather than a conserved antimicrobial function across vertebrates. Whether this embedded fragment can be endogenously processed and released from TNNT3 remains unresolved, because cleavage-site prediction was excluded in the present analysis. We further tested the antibacterial activity of the Bp Misgurin-like peptide. Unlike the native loach Misgurin, which has proved antibacterial activity without hemolysis, the Bp Misgurin-like peptide showed no detectable antibacterial activity against the six tested bacterial strains under the present assay conditions and concentration range. However, this result does not exclude possible activity against other microorganisms or under different experimental conditions. In fact, the native loach Misgurin was not examined in parallel under identical experimental conditions; it is used here as a functional reference rather than as a parallel positive control. Sequence comparison further showed that the codon corresponding to residue 2 is AAG in the three mudskippers and CAA in Ma, involving nucleotide differences at positions 4 and 6. Thus, relative to the Ma Misgurin, the Bp fragment contains a glutamine to lysine substitution at residue 2. Because lysine is cationic whereas glutamine is uncharged, this substitution increases rather than decreases the positive charge at this position. Therefore, the lack of detectable antibacterial activity in Bp cannot be explained simply by reduced positive charge at residue 2 [66,67], and may instead reflect other sequence-dependent physicochemical or membrane interaction properties.

Comparison of histone H2B sequences among representative vertebrates revealed high sequence conservation, while mudskippers showed a distinct glycine residue at position 66, where alanine was conserved in the other examined species. This pattern suggests a lineage-specific sequence variation within an otherwise highly conserved protein. These patterns suggest that AMP-derived genes may contribute to host defense diversity in amphibious habitats, although their ecological functions require experimental validation. Mudskipper adaptability also involves physiological [23], behavioral [26], and genetic [27] adaptations, rather than AMP-derived sequences alone.

The mudskipper genomes contained several clusters of AMP-derived genes, exhibiting chromosomal aggregation. Genome-wide mapping of AMP-derived genes in the three mudskipper species revealed clustered loci on several chromosomes, such as Chr12 and Chr14 in Bp. This pattern may indicate genomic regions with potential coordinated regulation or recombination, although expression and functional evidence are needed to test this possibility [68]. Genome-wide studies in fish have also shown that AMP-containing genes can be systematically identified and localized on assembled chromosomes, supporting the value of chromosomal mapping for interpreting AMP-derived repertoire organization [69]. Notably, histone-derived categories showed chromosomal clustering in the three mudskipper genomes, including Chr 12 and14 in Bp, Chr 14 and 19 in Pma, and Chr 8 and 21 in Pmo. This distribution pattern is reminiscent of histone gene aggregation in humans (Chr 1 and 6) and mice (Chr 3, 11, and 13) [70]. These histone-derived peptide candidates warrant more investigations through expression profiling and functional validation.

Several limitations of this study should be carefully considered. First, most of the identified AMP-derived sequences are computationally predicted candidates, and only the Bp Misgurin-like peptide was functionally tested in this study. Second, the five-genome comparison represents a broad rather than continuous aquatic-to-terrestrial comparison, which limits broader evolutionary interpretations. Third, the observed chromosomal clustering was based on genomic locations, without expression or regulatory evidence to assess coordinated regulation. Finally, the structural and physicochemical characteristics were primarily inferred from computational predictions. These limitations also highlight several directions for future research. Mudskipper-derived AMP candidates may have potential value for aquaculture applications, but their toxicity, antimicrobial spectrum, stability, and in vivo efficacy require systematic evaluation [5,7]. Future studies should also prioritize functional validation of AMP-derived genes, chemical synthesis of candidate peptides, and in vivo efficacy testing. Overall, this study improves our understanding of the diversity and phylogenetic evolution of putative AMP-derived sequences in amphibious fishes, and provides candidate resources for future investigation in medical and aquaculture applications.

## 5. Conclusions

Through genomic comparisons, this study identified 708 AMP-derived genes in three representative mudskipper species (Bp, Pma and Pmo), revealing fewer genes containing putative AMP-derived fragments than in zebrafish and humans. The smaller repertoire of putative AMP-derived genes in mudskippers may reflect multiple evolutionary processes, including gene gain or loss, altered duplication rates, genetic drift, and differences in genome assembly or annotation. Notably, while histone H2B exhibited high evolutionary conservation, mudskippers uniquely harbor a glycine substitution at position 66 (replacing alanine in other species). Interestingly, the corresponding Misgurin-like fragment was absent from the human *TNNT3* region. The Misgurin-like fragments of Bp, Pma, and Pmo contain lysine (K) at residue 2, whereas the native Ma Misgurin contains glutamine (Q) at the corresponding position. The Bp Misgurin-like peptide showed no detectable antibacterial activity under the tested conditions and concentration range, indicating that the observed activity difference cannot be simply attributed to the charge difference at this single residue. Furthermore, chromosomal aggregation of AMP-derived genes in mudskipper genomes suggests potential genomic organization of AMP-like sequence repertoires and provides candidate regions for future studies on regulation, recombination, and functional diversification. Overall, this study provides a comparative genomic framework for understanding the diversification of putative AMP-derived sequences in amphibious fishes, as well as offers a candidate resource for future functional validation and potential medical or aquaculture-related applications.

## Figures and Tables

**Table 1:** AMP categories and related AMP-derived gene numbers in the five examined vertebrates.

AMP Category	*D. rerio* (Dr)	*B. pectinirostris* (Bp)	*P. magnuspinnatus* (Pma)	*P. modestus* (Pmo)	*H. sapiens* (Hs)
Thrombin	80	64	58	78	84
Histone	124	27	35	31	59
Chemokine	46	15	15	28	41
BPTI	24	16	17	24	16
Scolopendin	18	14	18	24	20
Acipensin	14	12	9	15	13
β2M	22	5	2	6	19
Defensin	5	3	3	4	28
Hemoglobin	17	6	4	6	8
Ubiquitin	8	7	7	8	8
Neuropeptide	8	7	6	8	8
Lectin	5	7	2	9	2
Thymosin	5	2	4	5	6
Antiproteinase	4	3	2	2	6
GAPDH	2	3	3	2	2
Saposin-like	1	2	1	3	4
Hepcidin	1	2	1	5	1
PLA2	2	1	1	2	4
Ribonuclease	1	2	2	2	3
Skin-PYY	2	2	2	3	1
LEAP2	3	2	1	2	1
Amyloid	2	1	1	3	1
Peptide3910	1	2	2	2	1
Ubiquicidin	2	1	1	1	1
CcAMP1	1	1	1	1	1
Others	8	8	10	11	22
Total	406	215	208	285	360

## Data Availability

All data generated or analyzed for this study are included in this published article and its Supplementary files.

## Supplementary Materials

The following supporting information can be downloaded at: https://www.mdpi.com/article/10.3390/biology15161392/s1, Table S1: Antimicrobial activities of *Misgurnus anguillicaudatus* (Ma) Misgurin; Table S2: Hemolytic activities of *Misgurnus anguillicaudatus* Misgurin; Table S3: Comparison of physicochemical properties of Misgurin between Ma and Bp; Figure S1. Genome-wide distribution of AMP-derived genes in Pma. (a) The serial numbers at the top represent chromosome IDs. The haplotypic 25 chromosomes were assembled and annotated. Red bars represent histone-derived AMP-containing genes, while blue bars represent non-histone-derived AMP-containing genes. (b) A total of eight histone-derived AMP-containing genes are clustered on the Chr14. (c) Seven histone-derived AMP-containing genes are clustered on the Chr19. Figure S2. Genome-wide distribution of AMP-derived genes in Pmo. (a) The serial numbers at the top represent chromosome IDs. The haplotypic 23 chromosomes were assembled and annotated. Red bars represent histone-derived AMP-containing genes, while blue bars represent non-histone-derived AMP-containing genes. (b,c) Eight and twelve histone-derived AMP-containing genes are clustered on the Chr8 and the Chr21, respectively. Figure S3. Detailed sequences of TNNT3 gene in the human genome. The TNNT3 gene is located on the Chr11 (a), with 17 exons (b). Double slashes represent introns. Corresponding peptide sequence (e) is deduced from a target nucleotide sequence within the exons 16 and 17 (c,d; marked in purple and green, respectively). The red line marks an mRNA sequence for a special region corresponding to the Bp Misgurin-like fragment, while encoding a non-Misgurin peptide (e) due to loss of the target fragment with a different three-dimensional structure (f).

## References

[1] Patel S., Akhtar N. (2017). Antimicrobial peptides (AMPs): The quintessential ‘offense and defense’ molecules are more than antimicrobials. Biomed. Pharmacother..

[2] Tan P., Fu H.Y., Ma X. (2021). Design, optimization, and nanotechnology of antimicrobial peptides: From exploration to applications. Nano Today.

[3] Kawasaki H., Iwamuro S. (2008). Potential roles of histones in host defense as antimicrobial agents. Infect. Disord. Drug Targets.

[4] Athira P.P., Anju M.V., Anooja V.V., Archana K., Neelima S., Rosamma P. (2020). A histone H2A-derived antimicrobial peptide, Hipposin from mangrove whip ray, *Himantura walga*: Molecular and functional characterisation. 3 Biotech.

[5] Masso-Silva J.A., Diamond G. (2014). Antimicrobial peptides from fish. Pharmaceuticals.

[6] Bai Y.Q., Zhang W.B., Zheng W.B., Meng X.Z., Duan Y.Y., Zhang C., Chen F.Y., Wang K.J. (2024). A 14-amino acid cationic peptide Bolespleenin334-347 from the marine fish mudskipper *Boleophthalmus pectinirostris* exhibiting potent antimicrobial activity and therapeutic potential. Biochem. Pharmacol..

[7] Wang J.H., Su B.F., Dunham R.A. (2022). Genome-wide identification of catfish antimicrobial peptides: A new perspective to enhance fish disease resistance. Rev. Aquac..

[8] Chen X.Y., Yi Y.H., You X.X., Liu J.J., Shi Q. (2020). High-throughput identification of putative antimicrobial peptides from multi-omics data of the lined seahorse (*Hippocampus erectus*). Mar. Drugs.

[9] Shabir U., Ali S., Magray A.R., Ganai B.A., Firdous P., Hassan T., Nazir R. (2018). Fish antimicrobial peptides (AMPs) as essential and promising molecular therapeutic agents: A review. Microb. Pathog..

[10] Lata S., Sharma B.K., Raghava G.P.S. (2007). Analysis and prediction of antibacterial peptides. BMC Bioinform..

[11] Lee Y., Kim N., Roh H.J., Park J., Kim M., Lee J., Kim D.H. (2022). Hepcidin-1 in olive flounder (*Paralichthys olivaceus*): Gene expression, antimicrobial and therapeutic effects of synthetic peptides against bacterial and viral infections. Aquaculture.

[12] Katzenback B.A. (2015). Antimicrobial peptides as mediators of innate immunity in teleosts. Biology.

[13] Cole A.M., Weis P., Diamond G. (1997). Isolation and characterization of pleurocidin, an antimicrobial peptide in the skin secretions of winter flounder. J. Biol. Chem..

[14] Yi Y., Lv Y., You X., Chen J., Bian C., Huang Y., Xu J., Deng L., Shi Q. (2019). High throughput screening of small immune peptides and antimicrobial peptides from the Fish-T1K database. Genomics.

[15] Li Z., Hong W.S., Qiu H.T., Zhang Y.T., Yang M.S., You X.X., Chen S.X. (2016). Cloning and expression of two hepcidin genes in the mudskipper (*Boleophthalmus pectinirostris*) provides insights into their roles in male reproductive immunity. Fish Shellfish Immunol..

[16] You X.X., Bian C., Zan Q., Xu X., Liu X., Chen J., Wang J., Qiu Y., Li W., Zhang X. (2014). Mudskipper genomes provide insights into the terrestrial adaptation of amphibious fishes. Nat. Commun..

[17] Kurita M., Noma S., Ishimatsu A. (2021). Morphology of the respiratory vasculature of the mudskipper (Gobiidae: Oxudercinae). J. Morphol..

[18] Chen J., Nie L., Chen J. (2018). Mudskipper (*Boleophthalmus pectinirostris*) hepcidin-1 and hepcidin-2 present different gene expression profile and antibacterial activity and possess distinct protective effect against *Edwardsiella tarda* infection. Probiotics Antimicrob. Proteins.

[19] Ding F.F., Li C.H., Chen J. (2019). Molecular characterization of the NK-lysin in a teleost fish, *Boleophthalmus pectinirostris*: Antimicrobial activity and immunomodulatory activity on monocytes/macrophages. Fish Shellfish Immunol..

[20] Murdy E.O. (1989). A taxonomic revision and cladistic analysis of the Oxudercine gobies (Gobiidae: Oxudercinae). Rec. Aust. Mus. Suppl..

[21] Yi Y.H., You X.X., Bian C., Chen S., Lv Z., Qiu L., Shi Q. (2017). High-throughput identification of antimicrobial peptides from amphibious mudskippers. Mar. Drugs.

[22] Bian C., Huang Y., Li J., You X., Yi Y., Ge W., Shi Q. (2019). Divergence, evolution and adaptation in ray-finned fish genomes. Sci. China Life Sci..

[23] Liu H.H., Sun Q., Jiang Y.T., Fan M.H., Wang J.X., Liao Z. (2019). In-depth proteomic analysis of *Boleophthalmus pectinirostris* skin mucus. J. Proteom..

[24] Bian C., Huang Y., Li R., Xu P., You X., Lv Y., Ruan Z., Chen J., Xu J., Shi Q. (2024). Genomics comparisons of three chromosome-level mudskipper genome assemblies reveal molecular clues for water-to-land evolution and adaptation. J. Adv. Res..

[25] You X., Sun M., Li J., Bian C., Chen J., Yi Y., Yu H., Shi Q. (2018). Mudskippers and their genetic adaptations to an amphibious lifestyle. Animals.

[26] Huang Y., Li J., Bian C., Li R., You X., Shi Q. (2022). Evolutionary genomics reveals multiple functions of arylalkylamine N-acetyltransferase in fish. Front. Genet..

[27] Kim J., Lee C., Yoo D., Kim H. (2021). Genetic adaptations in mudskipper and tetrapod give insights into their convergent water-to-land transition. Animals.

[28] Wang D.D., Chen X., Zhang X., Li J., Yi Y., Bian C., Shi Q., Lin H., Li S., Zhang Y. (2019). Whole genome sequencing of the giant grouper (*Epinephelus lanceolatus*) and high-throughput screening of putative antimicrobial peptide genes. Mar. Drugs.

[29] Martin-Antonio B., Jiménez-Cantizano R.M., Salas-Leiton E., Infante C., Manchado M. (2009). Genomic characterization and gene expression analysis of four hepcidin genes in the redbanded seabream (*Pagrus auriga*). Fish Shellfish Immunol..

[30] Ghodsi Z., Kalbassi M.R., Farzaneh P., Mobarez A.M., Beemelmanns C., Amiri Moghaddam J. (2020). Immunomodulatory function of antimicrobial peptide EC-Hepcidin1 modulates the induction of inflammatory gene expression in primary cells of Caspian trout (*Salmo trutta caspius* Kessler, 1877). Fish Shellfish Immunol..

[31] Wang G., Li X., Wang Z. (2016). APD3: The antimicrobial peptide database as a tool for research and education. Nucleic Acids Res..

[32] Li R., Huang Y., Peng C., Gao Z., Liu J., Yin X., Gao B., Ovchinnikova T.V., Qiu L., Bian C. (2022). High-throughput prediction and characterization of antimicrobial peptides from multi-omics datasets of Chinese tubular cone snail (*Conus betulinus*). Front. Mar. Sci..

[33] Dong B., Yi Y.H., Liang L.F., Shi Q. (2017). High throughput identification of antimicrobial peptides from fish gastrointestinal microbiota. Toxins.

[34] McGinnis S., Madden T.L. (2004). BLAST: At the core of a powerful and diverse set of sequence analysis tools. Nucleic Acids Res..

[35] Hall T.A. (1999). BioEdit: A user-friendly biological sequence alignment editor and analysis program for Windows 95/98/NT. Nucleic Acids Symp. Ser..

[36] Yang Y., Yoo J.Y., Baek S.H., Song H.Y., Jo S., Jung S.H., Choi J.H. (2022). Chromosome-level genome assembly of the shuttles hoppfish, *Periophthalmus modestus*. GigaScience.

[37] Chen C., Wu Y., Li J., Wang X., Zeng Z., Xu J., Liu Y., Feng J., Chen H., He Y. (2023). TBtools-II: A “one for all, all for one” bioinformatics platform for biological big-data mining. Mol. Plant.

[38] Zhang M., Cao M., Xiu Y., Fu Q., Yang N., Su B., Li C. (2021). Identification of antimicrobial peptide genes in black rockfish Sebastes schlegelii and their responsive mechanisms to *Edwardsiella tarda* infection. Biology.

[39] Katoh K., Standley D.M. (2013). MAFFT multiple sequence alignment software version 7: Improvements in performance and usability. Mol. Biol. Evol..

[40] Beitz E. (2000). TeXshade: Shading and labeling of multiple sequence alignments using LaTeX2e. Bioinformatics.

[41] Edgar R.C. (2004). MUSCLE: Multiple sequence alignment with high accuracy and high throughput. Nucleic Acids Res..

[42] Tamura K., Stecher G., Kumar S. (2021). MEGA11: Molecular Evolutionary Genetics Analysis version 11. Mol. Biol. Evol..

[43] Rice P., Longden I., Bleasby A. (2000). EMBOSS: The European Molecular Biology Open Software Suite. Trends Genet..

[44] Gautier R., Douguet D., Antonny B., Drin G. (2008). HELIQUEST: A web server to screen sequences with specific α-helical properties. Bioinformatics.

[45] Abramson J., Adler J., Dunger J., Evans R., Green T., Pritzel A., Ronneberger O., Willmore L., Ballard A.J., Bambrick J. (2024). Accurate structure prediction of biomolecular interactions with AlphaFold 3. Nature.

[46] Yachdav G., Kloppmann E., Kajan L., Hecht M., Goldberg T., Hamp T., Hönigschmid P., Schafferhans A., Roos M., Bernhofer M. (2014). PredictProtein—An open resource for online prediction of protein structural and functional features. Nucleic Acids Res..

[47] Schrödinger L.L.C. (2024). The PyMOL Molecular Graphics System.

[48] Kent S.B.H. (1988). Chemical synthesis of peptides and proteins. Annu. Rev. Biochem..

[49] Jiang Z., Sun L., Li Y., Li H., Fu Y., Li J., Sun Z. (2025). The antimicrobial peptide D-CONGA-Q7 eradicates drug-resistant *Escherichia coli* by disrupting bacterial cell membranes. Biology.

[50] Papareddy P., Rydengård V., Pasupuleti M., Walse B., Mörgelin M., Chalupka A., Malmsten M., Schmidtchen A. (2010). Proteolysis of human thrombin generates novel host defense peptides. PLoS Pathog..

[51] Muñoz-Camargo C., Cruz J.C. (2024). From inside to outside: Exploring extracellular antimicrobial histone-derived peptides as multi-talented molecules. J. Antibiot..

[52] Hopo M.G., Mabrok M., Abu-Elala N., Yu Y. (2024). Navigating fish immunity: Focus on mucosal immunity and the evolving landscape of mucosal vaccines. Biology.

[53] Bird S., Tafalla C. (2015). Teleost Chemokines and Their Receptors. Biology.

[54] Das S., Pradhan C., Pillai D. (2022). β-Defensin: An adroit saviour in teleosts. Fish Shellfish Immunol..

[55] Bishop B.M., Juba M.L., Russo P.S., Devine M., Barksdale S.M., Scott S., Settlage R., Michalak P., Gupta K., Vliet K.A. (2017). Discovery of novel antimicrobial peptides from Varanus komodoensis (*Komodo dragon*) by large-scale analyses and de novo-assisted sequencing using electron-transfer dissociation mass spectrometry. J. Proteome Res..

[56] Shamova O.V., Orlov D.S., Balandin S.V., Shramova E.I., Tsvetkova E.V., Panteleev P.V., Leonova Y.F., Tagaev A.A., Kokryakov V.N., Ovchinnikova T.V. (2014). Acipensins—Novel antimicrobial peptides from leukocytes of the Russian sturgeon *Acipenser gueldenstaedtii*. Acta Naturae.

[57] Kieffer A.E., Goumon Y., Ruh O., Chasserot-Golaz S., Nullans G., Gasnier C., Aunis D., Metz-Boutigue M.H. (2003). The N- and C-terminal fragments of ubiquitin are important for the antimicrobial activities. FASEB J..

[58] Cho J.H., Sung B.H., Kim S.C. (2009). Buforins: Histone H2A-derived antimicrobial peptides from toad stomach. Biochim. Biophys. Acta Biomembr..

[59] Parseghian M.H., Luhrs K.A. (2006). Beyond the walls of the nucleus: The role of histones in cellular signaling and innate immunity. Biochem. Cell Biol..

[60] Doolin T., Gross S., Siryaporn A. (2020). Physical mechanisms of bacterial killing by histones. Adv. Exp. Med. Biol..

[61] Morgan M.J., Earnshaw J.C., Dhoot G.K. (1993). Novel developmentally regulated exon identified in the rat fast skeletal muscle troponin T gene. J. Cell Sci..

[62] Park C.B., Lee J.H., Park I.Y., Kim M.S., Kim S.C. (1997). A novel antimicrobial peptide from the loach, *Misgurnus anguillicaudatus*. FEBS Lett..

[63] Lorente-Martínez H., Agorreta A., Irisarri I., Zardoya R., Edwards S.V., San Mauro D. (2023). Multiple instances of adaptive evolution in aquaporins of amphibious fishes. Biology.

[64] Tennessen J.A. (2005). Enhanced synonymous site divergence in positively selected vertebrate antimicrobial peptide genes. J. Mol. Evol..

[65] Valenzuela C.A., Zuloaga R., Poblete-Morales M., Vera-Tobar T., Mercado L., Avendaño-Herrera R., Valdés J.A., Molina A. (2017). Fish skeletal muscle tissue is an important focus of immune reactions during pathogen infection. Dev. Comp. Immunol..

[66] Takada M., Ito T., Kurashima M., Matsunaga N., Demizu Y., Misawa T. (2023). Structure–activity relationship studies of substitutions of cationic amino acid residues on antimicrobial peptides. Antibiotics.

[67] Sitaram N., Chandy M., Pillai V.N., Nagaraj R. (1992). Change of glutamic acid to lysine in a 13-residue antibacterial and hemolytic peptide results in enhanced antibacterial activity without increase in hemolytic activity. Antimicrob. Agents Chemother..

[68] Zinani O.Q.H., Keseroglu K., Özbudak E.M. (2022). Regulatory mechanisms ensuring coordinated expression of functionally related genes. Trends Genet..

[69] Chen J., Zhang X., Li Y., Lv Y., You X., Shi Q., Wen Z. (2025). Chromosome-scale genome assembly and genome-wide identification of antimicrobial peptide-containing genes in the endangered long-finned gudgeon fish (*Rhinogobio ventralis*). Biology.

[70] Marzluff W.F., Gongidi P., Woods K.R., Jin J.P., Maltais L.J. (2002). The human and mouse replication-dependent histone genes. Genomics.

