# Supplementary material for: Comparative Genomics Reveals Diversification and Chromosomal Organization of Putative Antimicrobial Peptide-Derived Sequences in Amphibious Mudskippers
Source: Biology (Basel). 2026 Aug 14;15(16):1392. doi: 10.3390/biology15161392 (PMC13509821; doi:10.3390/biology15161392)
Supplement: Supplementary file 1 [file biology-15-01392-s001.zip › biology-4448185-supplementary.pdf]

Supplementary materials

Table S1. Antimicrobial activities of *Misgurnus anguillicaudatus* (Ma) Misgurin [62]

Microorganism	Minimal inhibitory concentrations (μM)
**Gram positive bacteria**	
Staphylococcus aureus	3.2
Streptococcus pneumoniae	3.2
**Gram negative bacteria**	
Escherichia coli	3.2
Salmonella typhimurium	3.2
**Fungi**	
Cryptococcus neoformans	3.2
Saccharomyces cerevisiae	1.6

Table S2. Hemolytic activities of *Misgurnus anguillicaudatus* Misgurin[62]

Concentrations (μM)	Hemolysis of sheep red blood cells (%)
2	0
4	0
10	23
20	23
40	46

Table S3. Comparison of physicochemical properties of Misgurin between Ma and Bp

**Parameter**	***B. pectinirostris*** **(Bp)**		***M. anguillicaudatus*** **(Ma)**	
Molecular weight	2501.97		2501.92	
Average Residue Weight	119.14		119.14	
Isoelectric Point	12.37		12.36	
Probability of expression in inclusion bodies	-0.61		0.02	
Charge	8.00		7.00	
**Property Residues**	**Number**	**Mole%**	**Number**	**Mole%**
Tiny (A+C+G+S+T)	6	28.57	6	28.57
Small (A+B+C+D+G+N+P+S+T+V)	7	33.33	7	33.33
Aliphatic (A+I+L+V)	5	23.81	5	23.81
Aromatic (F+H+W+Y)	1	4.76	1	4.76
Non-polar (A+C+F+G+I+L+M+P+V+W+Y)	7	33.33	7	33.33
Polar(D+E+H+K+N+Q+R+S+T+Z)	14	66.67	14	66.67
Charged (B+D+E+H+K+R+Z)	12	57.14	11	52.38
Basic (H+K+R)	10	47.62	9	42.86
Acidic (B+D+E+Z)	2	9.52	2	9.52

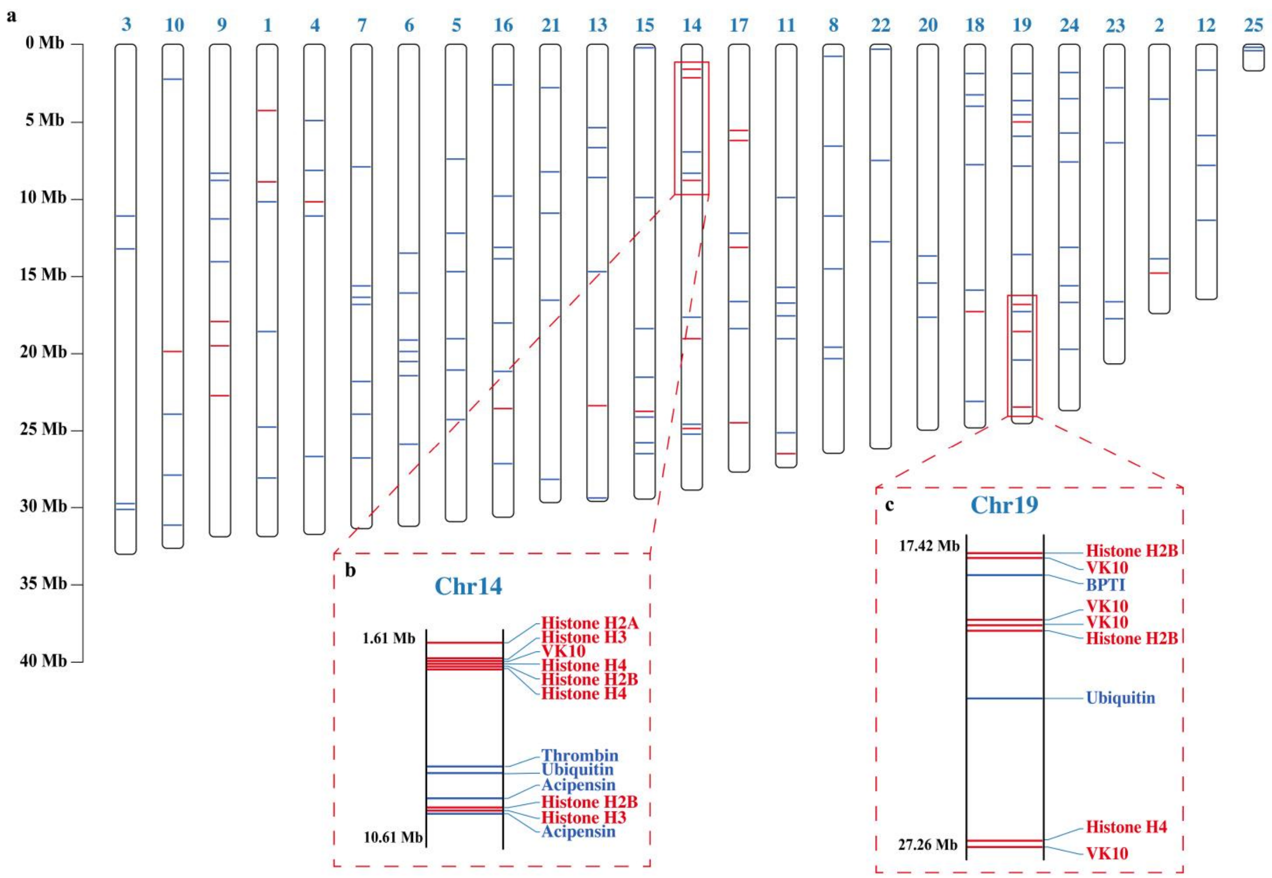

Figure S1. Genome-wide distribution of AMP-derived genes in Pma. (**a**) The serial numbers at the top represent chromosome IDs. The haplotypic 25 chromosomes were assembled and annotated. Red bars represent histone-derived AMP-containing genes, while blue bars represent non-histone-derived AMP-containing genes. (**b**) A total of eight histone-derived AMP-containing genes are clustered on the Chr14. (**c**) Seven histone-derived AMP-containing genes are clustered on the Chr19.

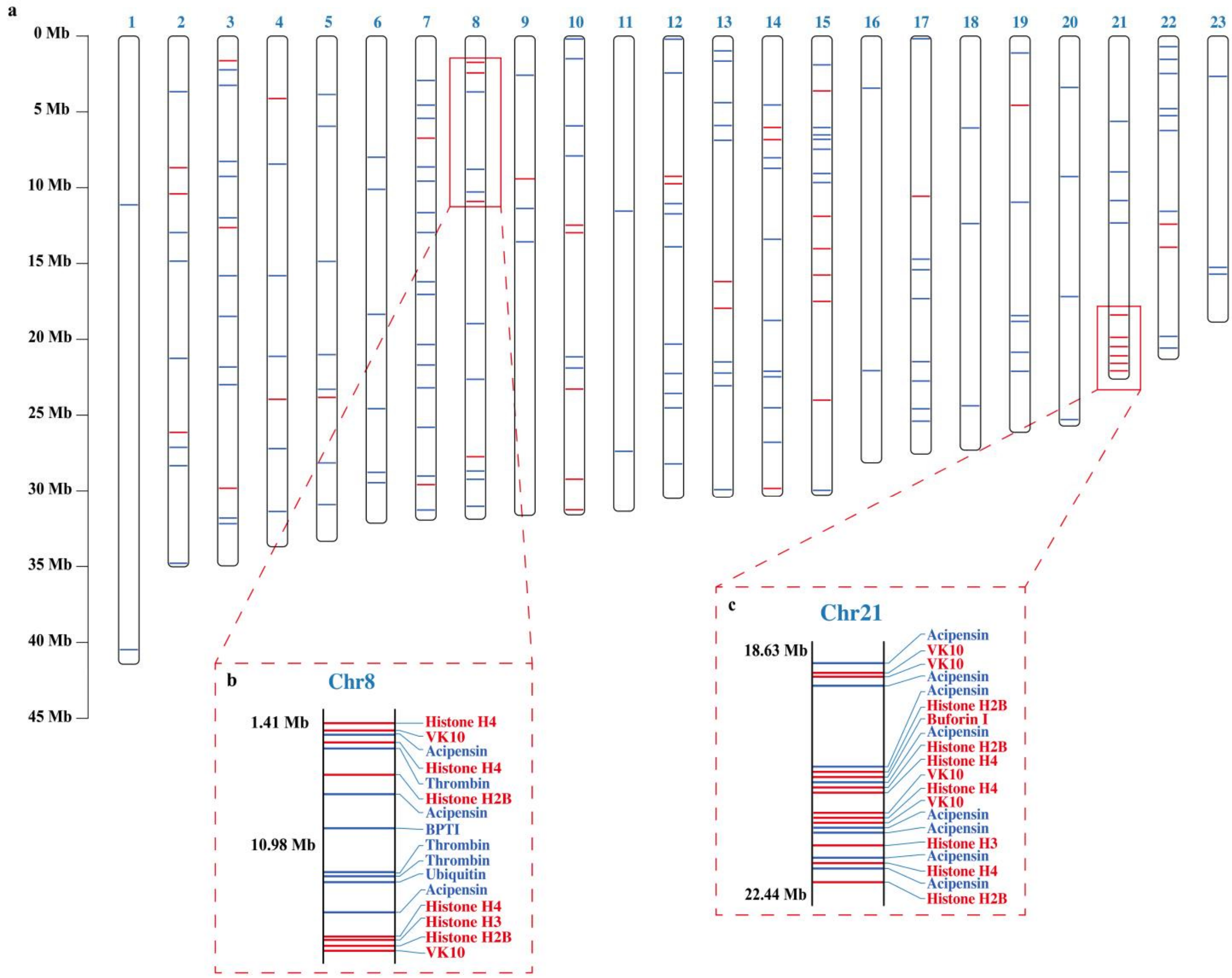

Figure S2. Genome-wide distribution of AMP-derived genes in Pmo. (**a**) The serial numbers at the top represent chromosome IDs. The haplotypic 23 chromosomes were assembled and annotated. Red bars represent histone-derived AMP-containing genes, while blue bars represent non-histone-derived AMP-containing genes. (**b-c**) Eight and twelve histone-derived AMP-containing genes are clustered on the Chr8 and the Chr21, respectively.

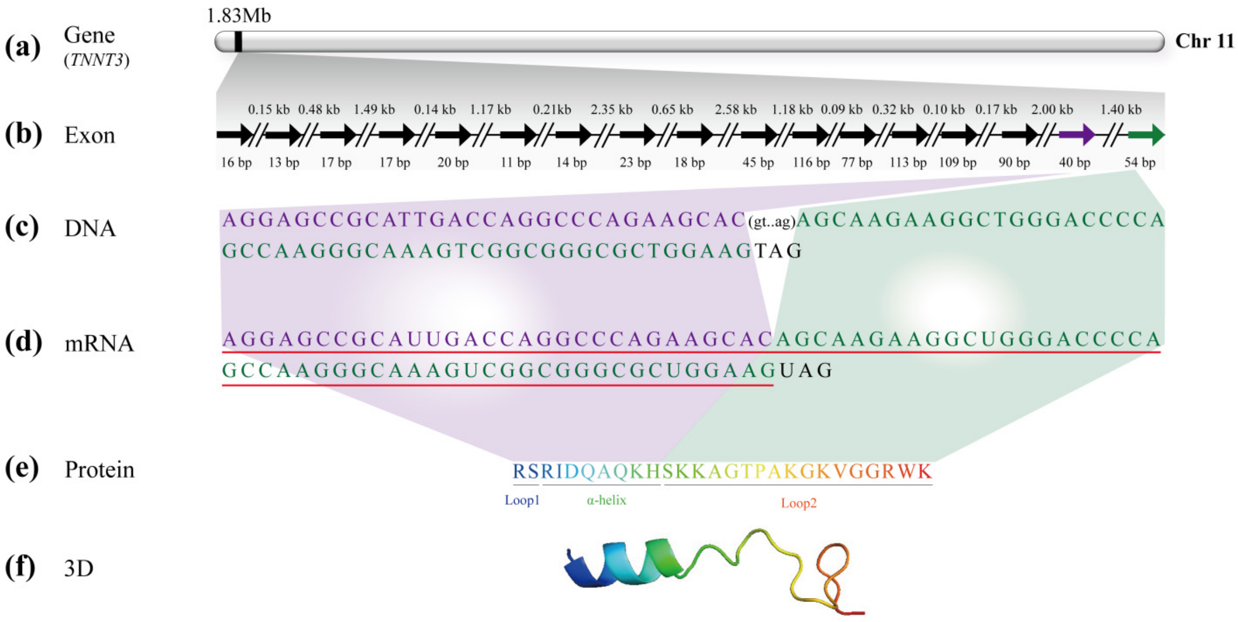

Figure S3. Detailed sequences of *TNNT3* gene in the human genome. The *TNNT3* gene is located on the Chr11 (**a**), with 17 exons (**b**). Double slashes represent introns. Corresponding peptide sequence (**e**) is deduced from a target nucleotide sequence within the exons 16 and 17 (**c-d**; marked in purple and green, respectively). The red line marks an mRNA sequence for a special region corresponding to the Bp Misgurin-like fragment, while encoding a non-Misgurin peptide (**e**) due to loss of the target fragment with a different three-dimensional structure (**f**).
